# Modulating biological aging with food-derived signals: a systems and precision nutrition perspective

**DOI:** 10.1038/s41514-025-00266-5

**Published:** 2025-08-20

**Authors:** Carsten Carlberg, Andreas Blüthner, Irini Schoeman-Giziakis, Annemarie Oosting, Luca Cocolin

**Affiliations:** 1 Institute of Animal Reproduction and Food Research, Polish Academy of Sciences, Olsztyn, Poland; 2 Institute of Biomedicine, University of Eastern Finland, Kuopio, Finland; 3 Dr. Blüthner & Partner, Bad Dürkheim, Germany; 4 Danone Global Research and Innovation, Utrecht, The Netherlands; 5 Cargill Food Solutions, Schipol, The Netherlands; 6 Department of Agricultural, Forest and Food Sciences, University of Torino, Torino, Italy

**Keywords:** Biomarkers, Nutrition

## Abstract

Lifespan extension has not prevented age-related decline. We propose that diet acts as a molecular modulator of aging, influencing inflammation, the microbiome, and systemic resilience. Biological age markers and AI-powered multi-omics reveal actionable dietary targets, including food-derived signals and Nutrition Dark Matter. We highlight precision nutrition and the EIT Food Healthy Aging Think & Do Tank as pathways to align science, policy, and practice for healthy aging.

## Introduction

Aging is a natural biological process that affects everyone but differently. While it cannot be stopped, increasing evidence suggests it can be modulated, and potentially reversed, through lifestyle and environmental interventions^1–3^. Among these, nutrition emerges as a major determinant of aging rate and resilience against non-communicable diseases (NCDs)^4,5^.

In 2023, life expectancy in the European Union averaged 81.4 years^6^. However, these additional years do not always correlate with good health. Indicators like Healthy Life Years (HLY) and Disability-Adjusted Life Years (DALY) highlight the gap between longevity and quality of life. Within the field of geroscience, the goal of healthy aging is not merely to extend lifespan but to maximize healthspan, the years lived in physical, emotional, and cognitive well-being^7^.

Lifestyle choices, including diet, physical activity, stress management, and sleep quality, significantly affect aging outcomes. However, the influence of genetic and epigenetic factors, as well as the microbiome, adds complexity to the equation. As researchers seek to understand what differentiates those who age healthily from those who develop chronic disease early, the integration of biological aging metrics offers a promising direction.

In addition to modifiable lifestyle factors, it is important to recognize that genetic background also contributes significantly to biological aging^8^. Studies on familial longevity and centenarian cohorts demonstrate that inherited factors can modulate aging trajectories, independently or synergistically with lifestyle inputs. Genome-wide association and sequencing studies suggest that longevity is partially heritable, with specific alleles linked to slower aging or disease resistance^9^. Importantly, the effectiveness of dietary or behavioral interventions may be influenced by an individual’s genetic makeup, underscoring the need for integrative approaches that consider both intrinsic and extrinsic drivers of biological aging.

Another emerging concept central to systems nutrition is that of Nutrition Dark Matter (NDM). This term encompasses the vast collection of over 139,000 food-derived small molecules, of which only a small fraction have known biological functions or identified protein targets^10^. These compounds may hold key regulatory roles in aging-related pathways but remain largely uncharacterized. As such, NDM offers a novel domain for discovery and a potential bridge between complex dietary patterns and specific molecular interventions aimed at modulating biological age.

This Perspective explores: i) how dietary patterns influence biological age *via* molecular mechanisms; ii) the role of the gut microbiome in mediating resilience; and iii) how omics and artificial intelligence (AI) enable precision nutrition for aging populations.

## Dietary patterns shape the biology of aging

Several factors, including diet, physical activity, and social relationships, influence aging and the development of NCDs. Research on centenarian populations suggests that plant-rich diets, balanced energy intake, and regular physical activity are linked to lower risks of chronic diseases and improved quality of life in older age^11^. These findings are further supported by recent evidence from two major prospective US cohorts (Nurses’ Health Study and Health Professionals Follow-up Study) involving over 100,000 participants followed for 30 years. These findings extend existing knowledge by linking diet adherence to multi-domain aging metrics, including cognitive and metabolic function. The studies showed that higher adherence to several dietary patterns, including AHEI (Alternative Healthy Eating Index), aMED (Alternative Mediterranean Index), DASH (Dietary Approaches to Stop Hypertension), and PHDI (Planetary Health Diet Index), was strongly associated with increased odds of achieving healthy aging^12^. This included preserved cognitive, physical, and mental function, as well as freedom from chronic diseases into older age. Among these, the AHEI showed the strongest association, nearly doubling the odds of healthy aging compared to the lowest adherence quintile.

While structured dietary patterns such as the Mediterranean diet or AHEI demonstrate robust associations with healthy aging, their effects emerge from the complex interplay of numerous food-derived bioactive compounds. Precision targeting of specific molecular pathways, for instance, those modulated by polyphenols, omega-3 fatty acids, or vitamin D3, can enhance our mechanistic understanding and intervention strategies. Rather than an either/or dichotomy, whole-diet frameworks and molecularly targeted approaches should be viewed as synergistic components of a unified strategy for healthy aging.

Beyond lifestyle factors, emerging research highlights the role of biological markers in assessing aging trajectories. One of the most promising tools in this area is the use of epigenetic clocks. Epigenetic factors, including immunomodulating micronutrients like vitamin D_3_, play a significant role in aging^13^. They support immune competence, reduce chronic inflammation, optimize metabolic pathways including management of oxidative stress, and help maintain a balanced gut microbiome, all of which contribute to overall health and longevity. Recognizing the interplay between lifestyle, social environment, and genetic predisposition is crucial for developing comprehensive strategies to support healthy aging at both individual and population levels^14^.

Most epigenetic clocks rely on bulk tissue samples such as blood, which contain varying cell types that shift with age. This heterogeneity can obscure true aging signals. It is thus important to distinguish between extrinsic clocks (influenced by cell composition) and intrinsic clocks (corrected for cell-type proportions). A clear distinction between chronological and biological age is essential for advancing aging research^15^. Epigenetic clocks, which estimate biological age based on DNA methylation patterns, can be broadly categorized into several types (Table 1). These are chronological clocks (e.g., Horvath^16^), which align closely with actual age; biological risk clocks (e.g., GrimAge^17^), which predict health outcomes and mortality risk; mitotic clocks (e.g., epiTOC2^18^), which track cellular replication; and noise barometer clocks, which capture stochastic variation in methylation patterns. Each category serves distinct purposes, from assessing disease risk to evaluating the impact of interventions, making the choice of clock crucial for accurately interpreting aging trajectories^17,19^. Moreover, recent research indicates that part of the variance in epigenetic clock rates may be genetically determined^20^.

**Table 1:** Comparison of aging clocks for an application in healthy aging research

Clock Type	Example(s)	Primary Target	Application in Healthy Aging
Chronological clock	Horvath^16^, Zhang^48^	Chronological age	Baseline age estimation, forensic science
Biological risk clock	GrimAge^17^, PhenoAge^49^	Disease risk, mortality	Nutritional intervention tracking
Mitotic clock	epiTOC2^18^	Cell division, cancer risk	Monitoring tissue turnover
Noise barometer clock	Outlier CpGs^50^	Stochastic epimutations	Biological instability, early cancer risk

The selection of a clock should align with the research objective. For instance, GrimAge is particularly well-suited for evaluating NCD risk and mortality prediction^17^. Since the introduction of the first epigenetic clock, the field has rapidly expanded, with numerous clocks now developed using diverse algorithms and data modalities^19^. Beyond epigenetics, aging clocks have also been constructed using taxonomic profiling of the gut microbiome, offering additional insights into host aging. Moreover, circadian rhythms within the gut epithelium are influenced by feeding patterns and microbiota-derived signals, which interact with host immune and neural systems^21^. This suggests that maintaining rhythmicity in microbiome-host interactions may be an additional target for interventions aimed at delaying cognitive aging. Further complexity arises from recent findings showing that plasma proteomic signatures can define organ-specific clocks in living individuals, opening new avenues for precision aging diagnostics^22^.

**Fig. 1:**
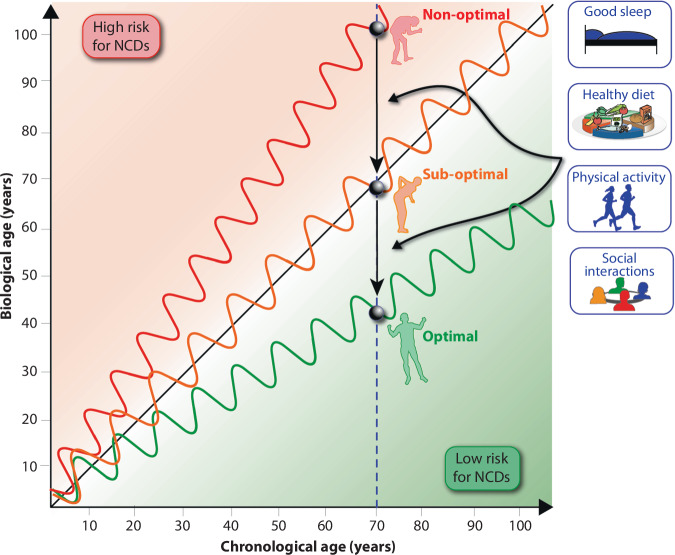
Diverging Trajectories of Biological Aging and Modifiable Lifestyle Interventions. Biological age, as opposed to chronological age, reflects the functional status and health risk of an individual. The diagonal dashed line represents chronological aging. Three illustrative aging trajectories, optimal (green), sub-optimal (orange), and non-optimal (red), demonstrate how biological age can diverge depending on lifestyle choices. Individuals aging optimally maintain a biological age below their chronological age and exhibit lower risk for NCDs, while those with non-optimal aging experience accelerated biological aging and higher NCD risk. Wave patterns represent short-term variability influenced by daily and seasonal factors. Arrows indicate how targeted interventions, such as good sleep, a healthy diet, regular physical activity, and social engagement, can decelerate aging and shift individuals toward a more favorable aging trajectory.

Figure 1 illustrates the concept of biological age and how it differs from chronological age in individuals. Chronological age refers to the actual age in years, while biological age reflects how well an individual’s body functions compared to others of the same chronological age. Factors such as genetics, lifestyle, and environmental influences contribute to this variation. In the figure, three hypothetical individuals are shown at the age of 70 (represented by circles). Despite having the same chronological age, their biological age varies. This is visually represented by three different trajectories:

- **Optimal aging** (green line): This individual’s biological age remains below chronological age, reflecting good health, a balanced diet, regular physical activity, and minimal chronic diseases.
- **Sub-optimal aging** (orange line): This individual’s experience moderate biological aging, possibly due to poor dietary habits or lack of physical activity, leading to early signs of aging and the onset of chronic health conditions.
- **Non-optimal aging** (red line): This individual’s biological age exceeds their chronological age, indicating accelerated aging, likely influenced by unhealthy lifestyle choices such as smoking or other forms of intoxication, poor diet, physical inactivity, and increased risk of age-related diseases like cardiovascular diseases or diabetes.

This emphasizes the role of lifestyle factors in influencing biological aging, with diet, exercise, sleep quality and social interactions serving as key factors in maintaining health and slowing down biological aging. This concept is critical to understanding how we can intervene to delay aging and prevent chronic diseases. Diet is a significant factor in influencing biological age. A balanced diet rich in nutrients, such as antioxidants, fiber, and polyunsaturated fats like omega-3 fatty acids, can help slow down the progression of aging. Conversely, poor dietary choices, such as high sugar, *trans*-fats, and low fiber, accelerate the aging process, increasing the risk of NCDs.

A plant-based diet containing high quality protein is associated with a 15% reduction in the risk of cardiovascular diseases and a 21% reduction in coronary artery disease^23,24^. Mechanistically, this is based on pathways like the inhibition of mTOR (mammalian target of rapamycin), modulation of sirtuins, and reduced oxidative stress. Moreover, adherence to the Mediterranean diet has been linked to reduced inflammation and improved cognitive function, with studies indicating a lower risk of cognitive decline and dementia among its adherents^25,26^. Vegan diets have been shown to improve glycemia and weight management for individuals with type 2 diabetes^24^. However, plant-based diets may lack essential micronutrients like vitamin B_12_, vitamin D_3_, iron, calcium, and long-chain omega-3 fatty acids. Ensuring adequate intake through careful dietary planning or supplementation is essential.

Furthermore, maintaining a healthy weight through a balanced diet and regular exercise can prevent or delay many chronic conditions, such as hypertension, diabetes, and heart disease. Interestingly, also social engagement and strong familial ties, as seen in Blue Zone populations, contribute significantly to longevity and overall well-being^27^. In contrast, loneliness has been associated with a poorer diet quality increasing the risk of malnutrition.

## The microbiome as a nutritional target for aging resilience

Human gut bacteria have co-evolved with their hosts over millions of years, achieving a level of adaptation that far exceeds that of most pathogens^28^. However, the dietary and environmental changes since the Industrial Revolution have placed new selective pressures on both the human genome and the gut microbiome. Due to their short generation times, microbes have adapted more rapidly, leading to marked interindividual variability in microbiome composition, shaped by diet, age, sex, geography, and health status.

The gut microbiome plays a critical role in immune development and maintains immune resilience and metabolic flexibility throughout life. It evolves from early colonization at birth, diversifies with diet and puberty, and stabilizes into a unique adult profile. Aging alters this balance, increasing variability and the abundance of taxa such as Bacteroides and Alistipes, influenced by lifelong dietary and lifestyle factors.

**Fig. 2:**
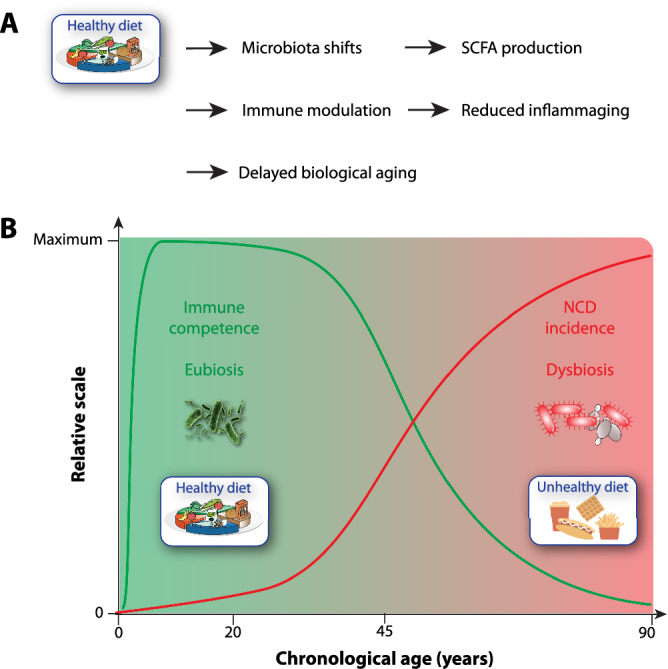
Impact of diet on the gut microbiome, immune function, and biological aging. **A** A healthy diet promotes beneficial microbiota shifts that enhance short-chain fatty acid (SCFA) production, modulate immune responses, and reduce chronic low-grade inflammation (“inflammaging”). These effects collectively contribute to a delay in biological aging. **B** Schematic overview of the age-related trajectories of immune competence and NCD incidence in relation to dietary quality. Early-life adherence to a healthy diet supports eubiosis and robust immune function (green curve), whereas an unhealthy diet is associated with dysbiosis, increased inflammation, and rising NCD burden over time (red curve). The transition zone reflects midlife vulnerability, where dietary choices critically shape aging trajectories. Illustrative icons depict examples of healthy (e.g., fiber-rich plant foods, fermented items) versus unhealthy (e.g., processed meats, sugary snacks) dietary patterns.

Human health can be actively shaped by modulating the microbiome through diet and broader food production practices. Diets like the Mediterranean diet are associated with microbial profiles protective against obesity-related cancers^29,30^. Although no universal “healthy microbiome” exists, certain taxa (e.g., Faecalibacterium) are linked to health benefits and lower epigenetic age^31^, while others (e.g., Ruminococcus) are associated with dysbiosis and higher risk for NCDs. A key mechanism behind this is the production of pro-inflammatory molecules, which drive systemic inflammation and disease progression^32^ (Fig. 2A). However, microbiome-based clocks remain population-specific and need further cross-cohort validation.

Food not only nourishes the body but also fuels gut microbes. High-quality dietary patterns, especially those rich in fiber, polyunsaturated fats, and plant-based foods, are robustly associated with improved systemic outcomes of aging. Specific dietary components, such as nuts, legumes, low-fat dairy, and unsaturated fats, are positively associated with multiple domains of healthy aging^12^. Thus, diet plays a central role in shaping microbial composition and activity. Long-term diets high in animal fat and protein are associated with inflammation, whereas fiber-rich, plant-based diets enhance the production of anti-inflammatory short-chain fatty acids. Microbiome-targeted nutrition is therefore central to promoting immune health and reducing NCD risk throughout life (Fig. 2B).

Autophagic clearance of bacterial toxins, damaged organelles, and misfolded proteins declines significantly with age^33^. This decline contributes to a vicious cycle: chronic gut inflammation impairs autophagy, which in turn exacerbates inflammation and promotes dysbiosis^34^. Nutritional interventions are therefore critical to restoring and maintaining healthy autophagic balance.

Preventing dysbiosis, or actively reshaping the gut microbiome, emerges as a key strategy for promoting healthy aging. Microbiota transplantation, for instance, offers the potential to transfer longevity-associated microbial profiles, with promising implications even in the context of neurodegenerative diseases^35,36^.

Furthermore, the gut serves as a central hub for neuro-immune communication. While early studies of the gut-brain axis emphasized the role of microbial metabolites in signaling to the central nervous system, recent findings reveal direct interactions between gut microbes and peripheral neurons. These insights support the idea that dietary modulation of the microbiome may offer protective effects against neurodegenerative diseases such as Alzheimer’s and Parkinson’s^37^. Food-based strategies for healthy aging, including cross-disciplinary and cross-sector collaborations, thus hold promise for delaying or preventing dementia.

Concurrently, aging-related disruptions in circadian clock communication, particularly between the central clock in the suprachiasmatic nucleus and peripheral tissues, have been linked to physiological decline^21^. These disruptions impact hormonal regulation (e.g., melatonin, cortisol), immune responses, metabolic rhythms, and cognitive performance. Interventions like light therapy and sleep hygiene can help resynchronize circadian timing and restore systemic balance.

Importantly, circadian rhythms in the gut epithelium are shaped by feeding patterns and microbiota-derived signals, which interface with host immune and neural systems^21^. Maintaining rhythmicity in microbiome-host interactions may therefore serve as an additional target for delaying cognitive aging. Time-restricted feeding, by aligning nutrient intake with internal circadian rhythms, represents a powerful intervention to re-establish rhythmicity in peripheral tissues.

To fully realize these opportunities, there is an urgent need to identify robust biomarkers that link microbiome composition to inflammatory status. Such markers could enable early, targeted interventions to reduce the risk of NCDs.

## AI and multi-omics enable precision aging interventions

Recent advancements in aging clocks provide a nuanced framework for applying biological age estimates to nutrition and health strategies. These technologies allow researchers to track aging biomarkers and identify interventions that may slow biological aging. The Biomarkers of Aging Consortium (www.agingconsortium.org) proposes a comprehensive framework for evaluating biological aging biomarkers across six critical dimensions: feasibility, validity, mechanism, generalizability, responsiveness, and cost. These criteria are crucial for determining whether biomarkers can transition from research tools to actionable instruments in clinical and public health settings. Emphasizing responsiveness to interventions, predictive validity for multiple age-related diseases, and robust cross-population validation enhances their translational utility^38^.

Omics technologies have also opened the door to precision nutrition, where diet can be tailored to an individual’s molecular profile, including genetics, epigenetics, metabolomics, lipidomics, and metagenomics, providing more effective strategies for promoting healthy aging. For example, studies have shown that bioactive nutrients and several dietary components like vitamin D_3_, polyphenols, and omega-3 fatty acids can modulate inflammatory pathways and improve metabolic function, which may help prevent chronic diseases associated with aging^13^.

To fully harness the potential of precision aging interventions, it is crucial to broaden our biochemical understanding of diet beyond established nutrients. As introduced earlier, the concept of NDM^10^ refers to a largely unexplored repertoire of food-derived small molecules, many of which lack known protein targets but may exert regulatory influence over key aging pathways. This chemical complexity rivals that of pharmaceutical libraries and includes molecules capable of modulating nearly half of the human proteome. Integrating NDM into systems biology frameworks holds strong potential for identifying novel dietary compounds that influence biological aging. For example, NDM profiling of fermented foods may reveal senotherapeutic polyphenol derivatives. The prioritization of NDM targets through omics data and aging clocks will help translate these discoveries into actionable nutritional interventions.

Emerging machine learning approaches, particularly deep learning models such as AltumAge^39^ or DeepMAge^40^ offer powerful tools for capturing complex, non-linear relationships between epigenetic markers of aging and for integrating diverse multi-omic datasets. When paired with explainable AI techniques like SHAP (Shapley Additive Explanations), these models yield interpretable insights into how specific DNA methylation features influence biological age predictions. Crucially, they can also help identify epigenetic loci that are responsive to lifestyle or dietary interventions, thereby supporting the development of precision nutrition strategies^41^.

More broadly, the integration of AI into aging research enhances our capacity to detect patterns in large-scale datasets, predict health outcomes, and design more targeted interventions for aging populations. For example, several microbiome-based aging clocks have already been established^42^. Specific microbial signatures have been shown to correlate with healthy aging and even predict survival^31^. This suggests that AI-powered models could also be instrumental in assessing the effectiveness of precision nutrition in promoting longevity.

Despite their promise, the translation of aging biomarkers remains limited so far. The non-linear trajectories of many molecular markers over the lifespan introduce further complexity^43^. To enable their integration into healthcare and personalized prevention strategies, additional efforts are needed to validate these biomarkers and ensure their interpretability and utility in real-world clinical settings.

- Biomarkers of aging should be validated in diverse populations and respond to modifiable interventions.
- The biomarkers must become cost-effective, interpretable, and clinically actionable, particularly when used as screening or response tools in personalized nutrition and preventive medicine.
- Implementation studies should link biomarkers to outcomes that impact how individuals “feel, function, and survive”.

A stepwise integration into clinical research (e.g., for stratifying trial participants) and then into practice (e.g., guiding lifestyle interventions or geroprotector trials) is recommended. Digital health tools and wearable sensors may help track biomarker-linked changes over time and build personalized aging profiles.

## The EIT food healthy aging think & do tank

To address the challenge of healthy aging on the levels addressed in the chapters above, EIT Food established in 2024 the HATDT^44^ as a proof-of-principle for transdisciplinary translation. The HATDT comprises over 50 experts from diverse countries across Europe, with backgrounds spanning academia, research, and industry (Supplementary Table 1). The initiative aims to tackle the complex issue of aging by integrating insights from multiple sectors, ensuring that the challenge is addressed comprehensively. Unlike standard stakeholder models, the HATDT applies reverse translational design, from community health needs to molecular strategies.

HATDT focuses on turning cutting-edge scientific knowledge into practical, scalable solutions for aging populations. It aims to uncover the key factors contributing to healthy aging and create pathways for longer, more healthy lives, focusing on dietary strategies, personalized nutrition, and microbiome health. The Think & Do Tank is structured around three strategic working groups, each focusing on different aspects of healthy aging research and intervention.

The collaborative efforts of the HATDT initiative emphasize the vital role of cross-sector partnerships in advancing our understanding and support of healthy aging. By bringing together research, policy, and community engagement, the initiative’s working groups aim to translate scientific insights into practical, scalable interventions tailored to the needs of aging populations. This model represents a comprehensive approach to healthy aging, one that integrates not only dietary strategies, but also physical activity, social connectivity, and technological innovation.

As a concrete example, HATDT has initiated a pilot program in collaboration with regional health authorities and local food producers to co-design and implement a dietary education and monitoring initiative for middle-aged individuals at risk of metabolic syndrome. This program incorporates biological age markers and community-based participatory principles to evaluate the efficacy of personalized dietary interventions. In parallel, HATDT is engaged in discussions with national policy stakeholders to integrate biological aging metrics into preventive nutrition guidelines. These actions reflect a commitment to reverse translation, from molecular evidence to community impact, and align with successful models in community-based personalized nutrition research^45^.

## Conclusion and call to action

This Perspective reframes food as a modular, multi-omic regulator of aging, not merely fuel or risk factor. By integrating food-derived compounds into a systems biology framework, we highlight new avenues for precision geroprevention. As populations age, preventive strategies must replace reactive care. Biological age offers a quantifiable, modifiable target, especially when paired with omics, AI, and aging clocks. The NDM framework emphasizes the need to map food bioactives and their molecular targets^10^. Just as the Human Genome Project revolutionized biomedicine, mapping the food–chemical–protein interface may similarly transform the field of precision nutrition. Linking such a foodome-based strategy to biological age markers holds strong potential for personalized dietary interventions that act on core aging pathways. In this way, NDM profiling of fermented foods may identify polyphenol derivatives with senotherapeutic activity^46^.

To translate these advances into meaningful change, we call for:

- **Advocate** for European-wide policies that ensure equitable access to nutritious food and age-supportive healthcare.
- **Fund** research into modifiable drivers of biological aging, particularly diet-microbiome-epigenome interactions.
- **Integrate** validated biomarkers into clinical and public health settings using cost-effective, interpretable platforms.
- **Expand** multisectoral partnerships, like the HATDT, to bridge innovation and community health, enhancing resilience.

By uniting data-driven approaches with accessible nutrition and social support systems, healthy aging can become the norm rather than the exception. Longitudinal evidence highlights the efficacy of structured dietary patterns in promoting multidimensional healthy aging. For example, diet-based biological age interventions in pre-diabetes patients could be performed using clock-responsive polyphenols^47^. Public health frameworks should incorporate these dietary patterns into age-supportive policies and nutritional guidance across Europe, as exemplified by existing national initiatives like the Nordic Nutrition Recommendations or the UK’s Eatwell Guide. Achieving this vision requires both scientific breakthroughs and systemic reforms, guided by biological age as a key metric for change.

The convergence of food science, molecular aging, and AI enables a new class of dietary interventions, grounded in biomarkers, personalized in action, and preventive in philosophy.

## Supplementary information

Supplementary Material

## Data Availability

No datasets were generated or analysed during the current study.

## References

[1] Mendoza-Nunez, V. M. & Mendoza-Soto, A. B. Is aging a disease? A critical review within the framework of ageism. *Cureus* **16**, e54834 (2024). 38405657 10.7759/cureus.54834 PMC10894070

[2] Carlberg, C., Ulven, S. M. & Velleuer, E. Aging: how science works. *Springer Textbook*, 10.1007/978-3-031-61257-2 (2024).

[3] Kennedy, B. K. et al. Geroscience: linking aging to chronic disease. *Cell* **159**, 709–713 (2014). 25417146 10.1016/j.cell.2014.10.039 PMC4852871

[4] Cohen, A. A. Complex systems dynamics in aging: new evidence, continuing questions. *Biogerontology* **17**, 205–220 (2016). 25991473 10.1007/s10522-015-9584-x PMC4723638

[5] Yang, J. H. et al. Loss of epigenetic information as a cause of mammalian aging. *Cell* **186**, 305–326.e327 (2023). 36638792 10.1016/j.cell.2022.12.027 PMC10166133

[6] Eurostat. https://ec.europa.eu/eurostat/statistics-explained/index.php?title=Mortality_and_life_expectancy_statistics.

[7] Dong, X., Milholland, B. & Vijg, J. Evidence for a limit to human lifespan. *Nature* **538**, 257–259 (2016). 27706136 10.1038/nature19793 PMC11673931

[8] Brooks-Wilson, A. R. Genetics of healthy aging and longevity. *Hum. Genet* **132**, 1323–1338 (2013). 23925498 10.1007/s00439-013-1342-z PMC3898394

[9] Erikson, G. A. et al. Whole-genome sequencing of a healthy aging cohort. *Cell* **165**, 1002–1011 (2016). 27114037 10.1016/j.cell.2016.03.022 PMC4860090

[10] Menichetti, G., Barabasi, A. L. & Loscalzo, J. Chemical complexity of food and implications for therapeutics. *N. Engl. J. Med* **392**, 1836–1845 (2025). 40334158 10.1056/NEJMra2413243 PMC12674684

[11] Guo, J. et al. Aging and aging-related diseases: from molecular mechanisms to interventions and treatments. *Signal Transduct. Target Ther.* **7**, 391 (2022). 36522308 10.1038/s41392-022-01251-0 PMC9755275

[12] Tessier, A. J. et al. Optimal dietary patterns for healthy aging. *Nat. Med.*10.1038/s41591-025-03570-5 (2025). 10.1038/s41591-025-03570-5 PMC12092270 40128348

[13] Carlberg, C. & Velleuer, E. Vitamin D and aging: central role of immunocompetence. *Nutrients* **16**, 398 (2024). 10.3390/nu16030398 PMC10857325 38337682

[14] Castruita, P. A., Pina-Escudero, S. D., Renteria, M. E. & Yokoyama, J. S. Genetic, Social, and Lifestyle Drivers of Healthy Aging and Longevity. *Curr. Genet Med Rep.* **10**, 25–34 (2022). 38031561 10.1007/s40142-022-00205-w PMC10686287

[15] Moqri, M. et al. Biomarkers of aging for the identification and evaluation of longevity interventions. *Cell* **186**, 3758–3775 (2023). 37657418 10.1016/j.cell.2023.08.003 PMC11088934

[16] Horvath, S. & Raj, K. DNA methylation-based biomarkers and the epigenetic clock theory of ageing. *Nat. Rev. Genet* **19**, 371–384 (2018). 29643443 10.1038/s41576-018-0004-3

[17] Teschendorff, A. E. & Horvath, S. Epigenetic ageing clocks: statistical methods and emerging computational challenges. *Nat. Rev. Genet.*10.1038/s41576-024-00807-w (2025). 10.1038/s41576-024-00807-w 39806006

[18] Yang, Z. et al. Correlation of an epigenetic mitotic clock with cancer risk. *Genome Biol.* **17**, 205 (2016). 27716309 10.1186/s13059-016-1064-3 PMC5046977

[19] Rutledge, J., Oh, H. & Wyss-Coray, T. Measuring biological age using omics data. *Nat. Rev. Genet* **23**, 715–727 (2022). 35715611 10.1038/s41576-022-00511-7 PMC10048602

[20] Miao, K. et al. Five years of change in adult twins: longitudinal changes of genetic and environmental influence on epigenetic clocks. *BMC Med.* **22**, 289 (2024). 38987783 10.1186/s12916-024-03511-y PMC11234599

[21] Mortimer, T., Smith, J. G., Munoz-Canoves, P. & Benitah, S. A. Circadian clock communication during homeostasis and ageing. *Nat. Rev. Mol. Cell Biol.*10.1038/s41580-024-00802-3 (2025). 10.1038/s41580-024-00802-3 39753699

[22] Oh, H. S. et al. Organ aging signatures in the plasma proteome track health and disease. *Nature* **624**, 164–172 (2023). 38057571 10.1038/s41586-023-06802-1 PMC10700136

[23] Dybvik, J. S., Svendsen, M. & Aune, D. Vegetarian and vegan diets and the risk of cardiovascular disease, ischemic heart disease and stroke: a systematic review and meta-analysis of prospective cohort studies. *Eur. J. Nutr.* **62**, 51–69 (2023). 36030329 10.1007/s00394-022-02942-8 PMC9899747

[24] Turner-McGrievy, G. & Harris, M. Key elements of plant-based diets associated with reduced risk of metabolic syndrome. *Curr. Diab Rep.* **14**, 524 (2014). 25084991 10.1007/s11892-014-0524-y

[25] Lourida, I. et al. Mediterranean diet, cognitive function, and dementia: a systematic review. *Epidemiology* **24**, 479–489 (2013). 23680940 10.1097/EDE.0b013e3182944410

[26] Petersson, S. D. & Philippou, E. Mediterranean diet, cognitive function, and dementia: a systematic review of the evidence. *Adv. Nutr.* **7**, 889–904 (2016). 27633105 10.3945/an.116.012138 PMC5015034

[27] Kreouzi, M., Theodorakis, N. & Constantinou, C. Lessons learned from blue zones, lifestyle medicine pillars and beyond: an update on the contributions of behavior and genetics to wellbeing and longevity. *Am. J. Lifestyle Med.* **18**, 750–765 (2024). 39507913 10.1177/15598276221118494 PMC11536469

[28] Berg, G. et al. Microbiome definition re-visited: old concepts and new challenges. *Microbiome* **8**, 103 (2020). 32605663 10.1186/s40168-020-00875-0 PMC7329523

[29] Fackelmann, G. et al. Gut microbiome signatures of vegan, vegetarian and omnivore diets and associated health outcomes across 21,561 individuals. *Nat. Microbiol.* **10**, 41–52 (2025). 39762435 10.1038/s41564-024-01870-z PMC11726441

[30] Almanza-Aguilera, E. et al. Mediterranean diet and olive oil, microbiota, and obesity-related cancers. From mechanisms to prevention. *Semin Cancer Biol.* **95**, 103–119 (2023). 37543179 10.1016/j.semcancer.2023.08.001

[31] Wilmanski, T. et al. Gut microbiome pattern reflects healthy ageing and predicts survival in humans. *Nat. Metab.* **3**, 274–286 (2021). 33619379 10.1038/s42255-021-00348-0 PMC8169080

[32] Hitch, T. C. A. et al. Microbiome-based interventions to modulate gut ecology and the immune system. *Mucosal Immunol.* **15**, 1095–1113 (2022). 36180583 10.1038/s41385-022-00564-1 PMC9705255

[33] Barbosa, M. C., Grosso, R. A. & Fader, C. M. Hallmarks of aging: an autophagic perspective. *Front. Endocrinol.* **9**, 790 (2018). 10.3389/fendo.2018.00790 PMC6333684 30687233

[34] Baechle, J. J. et al. Chronic inflammation and the hallmarks of aging. *Mol. Metab.* **74**, 101755 (2023). 10.1016/j.molmet.2023.101755 PMC10359950 37329949

[35] Novelle, M. G., Naranjo-Martinez, B., Lopez-Canovas, J. L. & Diaz-Ruiz, A. Fecal microbiota transplantation, a tool to transfer healthy longevity. *Ageing Res. Rev.* **103**, 102585. 10.1016/j.arr.2024.102585 (2025). 39586550 10.1016/j.arr.2024.102585

[36] Bruggeman, A. et al. Safety and efficacy of faecal microbiota transplantation in patients with mild to moderate Parkinson’s disease (GUT-PARFECT): a double-blind, placebo-controlled, randomised, phase 2 trial. *EClinicalMedicine* **71**, 102563. 10.1016/j.eclinm.2024.102563 (2024). 38686220 10.1016/j.eclinm.2024.102563 PMC11056595

[37] Herrera-Rincon, C., Murciano-Brea, J. & Geuna, S. Can we promote neural regeneration through microbiota-targeted strategies? Introducing the new concept of neurobiotics. *Neural Regen. Res.* **17**, 1965–1966 (2022). 35142677 10.4103/1673-5374.335149 PMC8848601

[38] Biomarkers of Aging, C. et al. Challenges and recommendations for the translation of biomarkers of aging. *Nat. Aging* **4**, 1372–1383 (2024). 39285015 10.1038/s43587-024-00683-3 PMC12262637

[39] Galkin, F., Mamoshina, P., Kochetov, K., Sidorenko, D. & Zhavoronkov, A. DeepMAge: a methylation aging clock developed with deep learning. *Aging Dis.* **12**, 1252–1262 (2021). 34341706 10.14336/AD.2020.1202 PMC8279523

[40] de Lima Camillo, L. P., Lapierre, L. R. & Singh, R. A pan-tissue DNA-methylation epigenetic clock based on deep learning. *npj Aging*10.1038/s41514-022-00085-y (2022).

[41] Bernal, M. C., Batista, E., Martínez-Ballesté, A. & Solanas, A. Artificial intelligence for the study of human ageing: a systematic literature review. *Appl. Intell.* **54**, 11949–11977 (2024).

[42] Chen, Y. et al. Human gut microbiome aging clocks based on taxonomic and functional signatures through multi-view learning. *Gut Microbes* **14**, 2025016. 10.1080/19490976.2021.2025016 (2022). 35040752 10.1080/19490976.2021.2025016 PMC8773134

[43] Shen, X. et al. Nonlinear dynamics of multi-omics profiles during human aging. *Nat. Aging* **4**, 1619–1634 (2024). 39143318 10.1038/s43587-024-00692-2 PMC11564093

[44] HATDT. www.eitfood.eu/projects/eit-food-healthy-ageing-think-tank.

[45] McCabe-Sellers, B. et al. Personalizing nutrigenomics research through community based participatory research and omics technologies. *OMICS* **12**, 263–272 (2008). 19040372 10.1089/omi.2008.0041

[46] Yang, F. et al. Effects of fermentation on bioactivity and the composition of polyphenols contained in polyphenol-rich foods: a review. *Foods* **12**, 3315 (2023). 10.3390/foods12173315 PMC10486714 37685247

[47] Naz, R. et al. Food polyphenols and type II diabetes mellitus: pharmacology and mechanisms. *Molecules* **28**, 3996 (2023). 10.3390/molecules28103996 PMC10222362 37241737

[48] Zhang, Y. et al. DNA methylation signatures in peripheral blood strongly predict all-cause mortality. *Nat. Commun* **8**, 14617 (2017). 28303888 10.1038/ncomms14617 PMC5357865

[49] Levine, M. E. et al. An epigenetic biomarker of aging for lifespan and healthspan. *Aging (Albany NY)* **10**, 573–591 (2018). 29676998 10.18632/aging.101414 PMC5940111

[50] Mei, X., Blanchard, J., Luellen, C., Conboy, M. J. & Conboy, I. M. Fail-tests of DNA methylation clocks, and development of a noise barometer for measuring epigenetic pressure of aging and disease. *Aging (Albany NY)* **15**, 8552–8575 (2023). 37702598 10.18632/aging.205046 PMC10522373

