# Supplementary material for: Modulating biological aging with food-derived signals: a systems and precision nutrition perspective
Source: NPJ Aging. 2025 Aug 20;11(1):76. doi: 10.1038/s41514-025-00266-5 (PMC12368197; doi:10.1038/s41514-025-00266-5)
Supplement: Supplementary file 1 — Supplementary Material [file 41514_2025_266_MOESM1_ESM.pdf]

Supplementary Material

Supplementary Table 1. Key Insights and Actions from the Three Working Groups of HATDT.

Working Group	Key Insights	Possible Actions
**1. Dietary Strategies for Preventing Age-Related NCDs**	- Investigate the impact of reducing salt, sugar, and saturated fats, while increasing fiber and polyunsaturated fats.<br>- Focus on bioactive nutrients, such as vitamin D, to reduce risks like hypertension, glucotoxicity, and lipotoxicity.<br>- Explore the role of chronic inflammation and deregulated nutritional signaling in NCD development.<br>- Understand that large-scale cohort data confirm that structured dietary patterns like AHEI and Mediterranean-style diets are predictive of successful aging.	- Educate stakeholders (healthcare professionals, consumers, food producers) on the link between diet, lifestyle, NCDs, and healthy aging.<br>- Promote dietary shifts to healthier options (e.g., whole grains, low-salt foods, plant-based proteins) while maintaining taste and appetite.<br>- Personalize nutrition by identifying interventions to reduce chronic inflammation, oxidative stress and strengthen immune health.<br>- Advocate for policies that promote access to healthy diets through educational programs.<br>- Use validated dietary pattern scores (e.g., AHEI, DASH) as educational tools to benchmark dietary quality in interventions or public awareness campaigns.
**2. Enhancing Health and Wellbeing Through Microbiome**	- Focus on preventing dysbiosis through diet, given the microbiome's high intra-individual variability.<br>- Utilize low-grade inflammation markers as indicators of microbiome balance.<br>- Balance the focus on protein with the importance of dietary fibers, which are essential in preventing dysbiosis.	- Conduct proof-of-concept studies to demonstrate the benefits of fiber, polyphenols, and probiotics to improve the health of humans and their microbiome (holobiont).<br>- Explore opportunities to enhance the circularity of the food system by valorizing waste streams from fruits and vegetables.<br>- Educate consumers on the impact of diet on gut health to encourage behavior change.<br>- Use effective communication strategies to deliver clear, engaging messages on microbiome health.
**3. Targeted Approaches for Healthy Aging**	- Identify barriers preventing individuals from adopting healthy choices, despite knowledge.<br>- Define "critical windows of change," when individuals are more likely to adopt healthy lifestyle practices.<br>- Analyze the impact of socio-environmental factors on sustained behavior change.	- Identify windows of opportunity when individuals are most motivated to change their lifestyle.<br>- Shape policies targeting populations most receptive to food-related behavioral changes.<br>- Educate healthcare professionals and policymakers on preventive techniques and support systems.<br>- Collaborate with industry to develop products that support a healthy diet across various life stages.
